# Hormonal Fluctuations and Periodontal Status in Postmenopausal Women

**DOI:** 10.1155/2022/9990451

**Published:** 2022-05-09

**Authors:** Mehrad Rafiei, Somayeh Salarisedigh, Parvin Khalili, Zahra Jamali, Farimah Sardari

**Affiliations:** ^1^Student Research Committee, Rafsanjan University of Medical Sciences, Rafsanjan, Iran; ^2^Department of Periodontics, School of Dentistry, Rafsanjan University of Medical Sciences, Rafsanjan, Iran; ^3^Department of Epidemiology, School of Public Health, Social Determinants of Health Research Center, Rafsanjan University of Medical Sciences, Rafsanjan, Iran; ^4^Non-Communicable Diseases Research Center, Rafsanjan University of Medical Sciences, Rafsanjan, Iran; ^5^Department of Oral Medicine, School of Dentistry, Rafsanjan University of Medical Sciences, Rafsanjan, Iran

## Abstract

**Introduction:**

While the short-term effects of hormonal events on gingival inflammation have been well described, their long-term effects on the periodontium have received less attention. Our investigation was aimed at evaluating the correlation between hormonal fluctuations and periodontal status in postmenopausal women from the profile of the Rafsanjan Cohort Study. *Material and Methods*. We used the data obtained from the profile of the Rafsanjan Cohort Study (RCS) as a part of the prospective epidemiological research studies in Iran (PERSIAN). The RCS includes 10,000 participants aged 35–70 years old. Among this population, the periodontal status data of 4143 women were available. Of these 4,143, the postmenopausal women were included in the study, and those who had a history of gingival treatment during the past 6 months were excluded from the study. Finally, 928 postmenopausal women were included in the present study. Periodontal status was assessed by measuring the clinical attachment loss, pocket depth, and bleeding on probing (BOP). Univariate and multivariate regression analyses were applied using three different models.

**Results:**

The results showed that 53.2% of postmenopausal women had periodontitis. There were significant differences between the participants with and without periodontitis in brushing frequency and educational status (*P* < 0.05). After adjusting for all potential confounders, no correlation was found between hormonal fluctuations and periodontal status.

**Conclusion:**

There was no correlation between hormonal fluctuations and periodontal status.

## 1. Introduction

Periodontal diseases are common oral infections that can destroy the tissues that support and surround the teeth and include gingivitis and periodontitis [1]. Periodontal diseases can be caused by pathogenic microbes present in the subgingival biofilm. The presence of these bacteria causes the production of chemokines and cytokines by the gingival epithelium, which increases the permeability of gingival capillaries and chemotaxis of polymorphonuclear neutrophils through the adhesive epithelium into the gingival sulcus [2]. This condition is often called gingivitis, which is characterized by bleeding, gingival swelling, and pain. If left untreated, the process can spread deep into the tissue, destroying the periodontal ligaments and alveolar bone. It allows the formation of periodontal pockets called periodontitis [3]. Periodontitis is a common disease with a prevalence rate of 17–82%. Its severe type has been reported to have a prevalence rate of 4–21% in different communities [4].

Periodontal diseases have also been shown to be associated with systemic diseases. Studies have shown an association between periodontitis and diabetes, cardiovascular disease, adverse pregnancy outcomes, chronic kidney disease, cognitive impairment, rheumatoid arthritis, and chronic obstructive pulmonary disease [5, 6].

Various factors such as stress, smoking, and diabetes are involved in periodontal diseases. Another factor associated with this disease is hormonal changes in women [7]. Studies have shown that puberty, menstrual cycle, pregnancy and its frequency, lactation, and menopause, which occur with hormonal changes in women, are associated with periodontitis [8]. These endogenous hormones are estrogen and progesterone [9]. An increase in gingivitis is associated with higher levels of these hormones due to the etiological role of sex steroids in altering the bacterial population of gingiva [10]. Androgens and estrogens have also been shown to be involved in the differentiation, proliferation, and growth of fibroblasts and keratinocytes [9]. In addition, androgens affect the differentiation and proliferation of osteoblasts. Furthermore, estrogen has been reported to play a role in the formation and maturation of connective tissue, inflammation of the gums, angiogenesis, reduction of keratinization, inhibition of inflammatory mediators and preinflammatory cytokines, stimulation of phagocytosis, and inhibition of chemotaxis of polymorphonuclear lymphocytes. Progesterone is also effective in inhibiting the fibroblast proliferation and collagen production, increasing prostaglandins, vasodilation, and permeability, minimizing the anti-inflammatory effect of glucocorticoids, and decreasing folate metabolism, which is essential in maintaining and regenerating the periodontal tissue [9, 11].

Therefore, it can be concluded that sex hormones have a regulatory role in the health of periodontal tissue and their changes can change a person's periodontal status. Therefore, further studies are required to investigate the effect of these hormonal changes on periodontal status. So far, many studies have examined hormone-dependent changes in women and shown the effect of hormonal changes during puberty, menstruation, pregnancy, and menopause on periodontal tissue [12–19], but each of these studies has examined each event separately during a short time. Therefore, this study was performed to evaluate the periodontal status in postmenopausal women aged 35 to 70 years in Rafsanjan, Iran. The hypothesis formulated in this study was that hormonal changes would affect the health of periodontal tissue.

## 2. Materials and Methods

This cross-sectional study was carried out based on the data of the Rafsanjan Cohort Study (RCS), which is a part of the prospective epidemiological research studies in Iran (PERSIAN) [20]. The RCS is conducted in Rafsanjan, one of the cities located in the southeast of Iran. The RCS includes 10,000 male and female participants aged 35–70 years old [21]. Among them, the periodontal status information of 4143 women was available. Of these 4,143 women, the postmenopausal women were included in the study, and those who had a history of gingival treatment during the past 6 months were excluded from the study. Finally, 928 postmenopausal women were included in the present study. This study was designed based on the PERSIAN cohort study protocol and was approved by the Ethics Committee of Rafsanjan University of Medical Sciences (Ethical codes: ID: IR.RUMS.REC.1400.094).

Information on hormonal fluctuations during the women's lifetime was collected through a self-administered questionnaire. All the questionnaires used in this study were prepared as a software program. The interviews were conducted face-to-face by trained interviewers. The participants' responses and the dental examination results were directly fed into the software. The validity and reliability of these questionnaires have been evaluated and confirmed [22]. The validity and reliability of the reproductive questionnaire have been investigated by Pezeshki et al. [23]. The content validity index (CVI) and content validity ratio (CVR) were calculated for each item. The minimum and maximum CVRs for all items were 0.80 and 1, respectively, so they were higher than the acceptance level (0.62). The total CVR for the whole questionnaire (average of the CVRs of all items) was 0.94. The minimum and maximum CVIs (average of CVIs for relevance, clarity, and simplicity criteria) were 0.80 and 0.96, respectively. The total CVI (average of CVIs of all items) was 0.91.

Based on a study by Romandini et al., the information of hormonal fluctuations during the women's lifetime was divided into two categories: endogenous and exogenous events[24].

Endogenous events included menarche age, menopause age, reproductive period, type of menopause, age at first delivery, age at first pregnancy, number of pregnancies, number of abortions, breastfeeding duration (total), and history of infertility. Exogenous events consisted of the use of oral contraceptives (OCP), hormone replacement therapy, and infertility-related medications. All variables related to hormonal fluctuations were divided into quartiles for statistical analysis.

The covariables of the study were also collected based on the findings of other studies [25–29] and the questions in the RCS questionnaire. These variables included age, educational status, body mass index (BMI), history of cigarette/hookah smoking, history of hypertension and diabetes, wealth index score, marital status, and frequency of brushing.

The periodontal status of participants was assessed by clinical attachment loss, pocket depth, and bleeding on probing (BOP). The measurements were performed by the walking probing method using the Williams Probe (Williams coded, Hu-Friedy, USA, Michigan ”o” probe). The clinical examination included examination of all permanent, completely erupted teeth except all the erupted third molars. The obtained CAL was the sum of the probing depth and gingival recession. When CAL progression was ≥1 mm or pocket depth was >3 mm with BOP in ≥2 teeth, it was defined as periodontitis [30].

Categorical data across hormonal changes were analyzed using the chi-square test. A *T*-test was used to compare continuous variables between hormonal fluctuation groups. Logistic regression models were used to determine the association between lifetime hormonal fluctuations and periodontitis incidence. Confounders were identified using relevant epidemiological literature. To this end, separate bivariate models were run to detect the variables associated with periodontitis.

Three models in the regression analysis were used. Moreover, the baseline model (crude model) was stratified on the condition of lifetime hormonal fluctuations. In addition, the adjusted model 1 included age, and model 2 had a further adjustment of the variables related to the lifestyle (smoking cigarettes and hookah), history of diabetes (yes/no), history of hypertension (yes/no), and body mass index (BMI) (continuous variable), as well as WSI (continuous variable). The adjusted model 3 had an additional adjustment for brushing frequency. All analyses were performed by STATA software (Version 14). All *p* values were two-sided.

## 3. Results

In the present study, among 928 participants in the baseline data collection phase of the RCS, 494 (53.23%) had periodontitis (defined as CAL≥1 mm or pocket depth >3 with BOP) and 434 (46.77%) did not have periodontitis.

The demographic characteristics of participants, including age, BMI, WSI, marital status, history of hypertension, number of lost teeth, tooth brushing frequency, educational status, history of diabetes, history of gestational hypertension and gestational diabetes, cigarette/hookah smoking, and variables related to hormonal fluctuations are shown in Table 1. There were significant differences between the two groups in brushing frequency and educational status (*P* > 0.05) (Table 1).

Table 2 shows the association between hormonal fluctuations and periodontitis incidence using the crude and three adjusted models. There was also no significant association between the incidence of periodontitis and type and age of menopause, number of pregnancies, use of OCP, taking HRT drugs, history of infertility and hysterectomy, use of infertility drugs, menstruation age, reproductive period, number of abortions and pregnancies, breast feeding duration, age at first pregnancy, and age at first childbirth.

## 4. Discussion

In the present study, the null hypothesis concerning the lack of correlation between hormonal fluctuations during women's lifetime and periodontal status was confirmed. There was a significant relationship between the patients with and without periodontitis in brushing frequency and educational status.

Similar to the present study, it has been shown that sociodemographic status can affect the oral health of pregnant women, and oral hygiene during this period can eliminate the adverse effects on pregnancy [31].

We were not able to find any other epidemiological population-based studies on the relationship between the history of endogenous hormonal fluctuations and periodontitis except two studies by Yakar et al. [32] and Romandi et al. [24] Romandi et al. showed periodontitis was directly associated with a longer duration of breastfeeding and was inversely associated with artificial menopause and early and late menopausal ages. The difference between the present study and Romandi et al.'s study can be due to differences in race, group classification, sample size, and the use of different models. They evaluated the periodontal status by the community periodontal index (CPI), which examines the periodontal status by pocket depth. It is possible to record increasing probing depths without the loss of clinical attachment if they have been caused by the development of pseudopockets [33]. Conversely, disease progression (loss of clinical attachment) can occur in the absence of deepened probing depths, [34] but CAL, which is also measured using the periodontal probe, represents the extent of periodontal support lost around a tooth and the distance from the cementoenamel junction (CEJ) to the base of the pocket [35].

Similar to the present study, Yakar et al. [32] showed that events with hormonal changes were not associated with periodontitis and the presence of a depressive mood in different periods in a women's lifetime could be a risk factor for periodontitis.

Numerous studies have shown that pregnancy can cause periodontal diseases as well as tooth decay and loss [36–38]. Increased CAL has been shown to cause an active periodontal infection that is accelerated during pregnancy [36]. Laine reported changes in salivary composition in late pregnancy and during lactation which might temporarily predispose a woman to dental caries and erosion. Although their underlying pregnancy-related changes in the oral environment may have some untoward temporary or permanent effects on oral health [39]. On the other hand, two cross-sectional cohort studies declared that pregnancy is not a risk factor for periodontitis [24, 32]. Laine et al. indicated most of these effects could be avoided by practicing good oral hygiene.

Various studies have shown the effect of hormonal changes on the periodontal status of women in different periods of life. It has been shown that hormonal changes in the menstrual cycle alone cannot have any specific clinical effect on the periodontal tissue, although this effect is more noticeable in individuals with gingivitis [39]. Furthermore, a cross-sectional study in a Portuguese population showed that menopause could cause periodontal diseases and the hormone replacement therapy (HRT) could improve periodontal status in postmenopausal women [40]. Romandi et al. also found that early and late menopause age is a risk factor for periodontitis [24].

Some studies have reported a correlation between systemic osteoporosis and alveolar bone loss. They have also shown that decreased bone marrow density of alveolar bone can lead to attachment loss and tooth loss [41, 42]. However, similar to the present study, Yakar et al. reported no correlation between menopause age and periodontitis. They also declared that osteoporosis is not a risk factor for periodontitis [32]. Pan et al. also reported that oral hygiene plays an important role in periodontitis even more than bone mineral density [43]. Studies have also shown that a depressive mood is more common in women of menopausal age, and these women are more likely to have oral problems, so menopause itself cannot be a risk factor [32, 44].

In a case-control study, prolonged breastfeeding increased some of the periodontal indices, including pocket depth and BOP, but in the present study, there was no significant difference between periodontitis and the duration of breastfeeding [45]. Studies have also examined the exogenous hormonal changes [46–48]. Prachi et al. showed taking OCP can be associated with periodontitis, [46] while Preshaw reported OCP should not be viewed as a risk factor for gingival or periodontal diseases [47]. A cohort study in South Korea showed that HRT can play a significant role in the occurrence of periodontal diseases [48]. Yet, in line with the results of the present study, Pizzo et al. found that HRT was not associated with clinical attachment level and pocket depth [49]. Regarding the other variables studied, the findings of the present study were similar to those of other studies [24, 32, 39, 49]. However, it should be noted that due to differences in the study design, it is not possible to compare the present study with other studies.

Differences between the results of the present study and those of other studies may be due to differences in the study design, demographic characteristics, ethnicity, diet, geographical location, cultural variables, and different criteria for the diagnosis of periodontitis. The extent and severity of periodontal diseases were not considered in the present study. On the other hand, the lack of a single definition of periodontal diseases makes them difficult to compare with the existing studies. On the other hand, there is no single variable that fully expresses the full complexity of periodontal disease progression. The average CAL is an indication of the severity of the disease, while the number of teeth with envelopes indicates its extent; however, the loss of adhesion is considered the “gold standard” in diagnosis. In the present study, based on the American Academy of Periodontology, [30] CAL progression ≥1 mm or pocket depth >3 mm with BOP in ≥2 teeth was defined as periodontitis. However, Romandi et al. [24] defined periodontitis based on the community periodontal index (CPI), and Yakar et al. [32] only considered CAL for periodontitis diagnosis.

The strengths of the present study include the large sample size and comprehensive evaluation of various factors involved in periodontal status among women. Therefore, a major strength of the present research is the adjustment of different potential confounders such as demographic information, history of diseases, and reproductive characteristics. This study also had its limitations. Hormonal fluctuations were self-reported by the participants. Hence, the obtained information might not be complete for reasons such as the passage of time, inaccuracy, etc. Yet, it is worth mentioning that people who have diseases related to memory disorders, such as Alzheimer's disease, were excluded from the PERSIAN cohort study [20].

## 5. Conclusion

Within the limitations of the study, including the use of a self-reported questionnaire and considering the retrospective aspects of the present study, the findings showed no correlation between hormonal fluctuations and periodontal status. Longitudinal studies are needed to corroborate the temporality of the association between variables. Future studies using similar periodontitis diagnostic criteria are recommended to evaluate the effects of hormone-dependent events in different communities.

## Figures and Tables

**Table 1:** Characteristics of the study sample according to the periodontal status (*n* = 928).

Variable	Overall (*n* = 928)	No periodontitis (*n* = 434)	Periodontitis (*n* = 494)	*P*value
Age (year), mean (SD)	53.98, (7.58)	53.60, (8.27)	54.30, (6.90)	0.158
Number of lost teeth, mean (SD)	11.72, (6.62)	11.51, (7.26)	11.91, (6.01)	0.352
BMI (Kg/M^2^), mean (SD)	29.69, (4.80)	29.58, (4.62)	29.79, (4.97)	0.501
Wealth index score, mean (SD)	−0.0974, (0.97)	−0.0678, (0.98)	−0.123, (0.96)	0.384
Marital status, *N*[%]				0.948
Never married	11, (1.2)	5, (1.1)	6, (1.2)	
Married and living with a spouse	788, (84.9)	367, (84.5)	421, (85.2)	
Married but living alone	129, (13.9)	62, (14.3)	67, (13.5)	
History of hypertension, *N*[%]				0.407
No	565, (61.1)	270, (62.5)	295, (59.8)	
Yes	360, (38.9)	162, (37.5)	198, (40.1)	
Tooth brushing frequency, *N*[%]				0.014
≤1 per week	92, (10.3)	34, (8.3)	58, (11.9)	
2–6 per week	288, (32.2)	119, (29.1)	169, (34.9)	
≥1 per day	512, (57.4)	255, (62.5)	257, (53.1)	
Educational status, *N*[%]				0.011
None	114, (12.2)	48, (11)	66, (13.36.3)	
1–5 years	329, (35.4)	134, (30.9)	195, (39.47)	
6–12 years	386, (41.5)	199, (51.5)	187, (48.4)	
More than 12	99, (10.6)	53, (12.2)	46, (9.31)	
History of diabetes, *N*[%]				0.407
Yes	325, (35)	158, (36.4)	167, (33.8)	
No	603, (65)	276, (63.5)	385, (66.1)	
Cigarette/Hookah smoking, *N*[%]				0.956
Smokers	66, (7.1)	31, (7.2)	35, (7.1)	
Nonsmokers	858, (92.9)	400, (92.8)	458, (92.9)	
Infertility *N*[%]				0.939
Yes	146, (15.8)	69, (15.9)	77, (15.7)	
No	778, (84.1)	365, (84.1)	413, (84.3)	
History of infertility drug use, *N*[%]				0.783
Yes	51, (5.6)	23, (5.3)	28, (5.5)	
No	873, (94.4)	411, (94.7)	462, (94.5)	
Use of oral contraceptives, *N*[%]				0.344
Yes	413, (44.5)	186, (42.8)	227, (45.9)	
No	515, (55.5)	248, (57.1)	267, (54)	
Taking hormonal replacement drugs, *N*[%]				0.722
Yes	55, (5.9)	27, (6.2)	28, (5.7)	
No	873, (94.1)	407, (93.8)	466, (94.3)	
Menopause age, *N*[%]				0.360
≤45	274, (28.72)	105, (27.93)	169, (29.24)	
46–50	316, (33.12)	132, (35.11)	184, (31.83)	
51-52	159, (16.67)	54, (14.36)	105, (18.17)	
≥53	205, (21.49)	85, (22.61)	120, (21.49)	
Reproductive period, *N*[%]				0.454
≤32	273, (29.4)	121, (27.9)	152, (30.8) 138, (23.88)	
33–36	239, (25.7)	118, (27.2)	121, (24.5)	
37–39	233, (25.11)	115, (26.5)	118, (23.9)	
≥40	183, (19.7)	80, (18.4)	103, (20.8)	
Breast feeding duration, *N*[%]				0.573
≤44	231, (25.5)	108, (25.5)	123, (25.5)	
45–73	226, (25)	111, (26.2)	115, (23.9)	
74–108	239, (26.4)	103, (24.3)	136, (28.2)	
≥109	209, (23.1)	101, (23.9)	108, (22.4)	
Number of pregnancies, *N*[%]				0.098
≤4	361, (39.1)	174, (40.1)	187, (38.2)	
5	147, (15.9)	77, (17.5)	71, (14.5)	
6-7	245, (26.5)	99, (22.8)	146, (29.8)	
≥8	171, (18.5)	85, (19.6)	86, (17.5)	
Number of abortions, *N*[%]				0.613
0	562, (62.1)	259, (61.2)	303, (62.9)	
≥1	343, (37.9)	164, (38.8)	179, (37.1)	
Age at first living childbirth, *N*[%]				0.806
≤18	242, (27)	106, (25.4)	136, (28.3)	
19-20	212, (23.6)	101, (24.2)	111, (23.1)	
21–23	244, (27.2)	115, (27.6)	129, (26.9)	
≥24	199, (22.2)	95, (22.8)	104, (21.7)	
Age at first pregnancy, *N*[%]				0.868
≤17	232, (25.6)	106, (25.1)	126, (26.1)	
18–20	314, (34.7)	143, (33.8)	171, (35.5)	
21-22	160, (17.7)	78, (18.4)	82, (17)	
≥23	199, (22)	96, (22.7)	103, (21.3)	
Menarche age, *N*[%]				0.664
≤12	235, (25.3)	111, (25.6)	124, (25.1)	
13	234, (25.2)	111, (25.6)	123, (24.9)	
14-15	386, (41.6)	183, (42.2)	203, (41.1)	
≥160	73, (7.9)	29, (6.7)	44, (8.9)	

**Table 2:** Crude and adjusted ORs (95% CIs) for the prevalence of periodontitis (CAL ≥1 mm) according to different lifetime hormonal fluctuation events.

Variable	Crude, OR (95% CI)	Adjusted model 1, OR (95% CI)	Adjusted model 2, OR (95% CI)	Adjusted model 3, OR (95% CI)
Type of menopause				
Natural	1.07, (0.80–1.44)	1.05, (0.78–1.41)	1.05, (0.78–1.42)	1.02, (0.75–1.39)
Artificial	1, (ref)	1, (ref)	1, (ref)	1, (ref)
Hysterectomy				
Yes	0.97, (0.72–1.30)	0.98, (0.73–1.32)	0.99, (0.73–1.33)	1.03, (0.76–1.41)
No	1, (ref)	1, (ref)	1, (ref)	1, (ref)
Infertility				
Yes	0.98, (0.69–1.40)	1.00, (0.70–1.43)	1.02, (0.71–1.46)	0.98, (0.77–1.42)
No	1, (ref)	1, (ref)	1, (ref)	1, (ref)
History of infertility drug use				
Yes	1.08, (0.61–1.90)	1.10, (0.62–1.94)	1.08, (0.61–1.92)	1.14, (0.63–2.09)
No	1, (ref)	1, (ref)	1, (ref)	1, (ref)
OCP use				
Yes	1.13, (0.87–1.46)	1.12, (0.86–1.65)	1.12, (0.86–1.46)	1.10, (0.84–1.44)
No	1, (ref)	1, (ref)	1, (ref)	1, (ref)
History of hormonal replacement drug use				
Yes	1.10, (0.61–1.75)	1.12, (0.64–1.93)	1.12, (0.65–1.94)	1.24, (0.70–2.18)
No	1, (ref)	1, (ref)	1, (ref)	1, (ref)
Menopause age				1, (ref)
≤45	1, (ref)	1, (ref)	1, (ref)	1, (ref)
46–50	0.91, (0.65–1.27)	0.89, (0.64–1.25)	0.89, (0.64–1.24)	0.91, (0.65–1.29)
51–52	1.30, (0.87–1.95)	1.24, (0.82–1.87)	1.24, (0.82–1.88)	1.14, (0.75–1.75)
≥53	0.78, (0.54–1.14)	0.75, (0.51–1.09)	0.75, (0.81–1.02)	0.78, (0.53–1.16)
Reproductive period				
≤32	1, (ref)	1, (ref)	1, (ref)	1, (ref)
33–36	0.81, (0.57–1.15)	0.80, (0.51–1.13)	0.78, (0.54–1.11)	0.77, (0.51–1.12)
37–39	0.81, (0.57–1.15)	0.75, (0.55–1.12)	0.78 (0.55–1.12)	0.80, (0.56–1.16)
≥40	1.02, (0.70–1.49)	0.97, (0.66–1.43)	0.98, (0.66–1.44)	0.97, (0.65–1.44)
				1.04, (0.70–1.55)
Breast feeding duration				
≤44	1, (ref)	1, (ref)	1, (ref)	1, (ref)
45–73	0.90, (0.63–1.31)	0.91, (0.63–1.32)	0.93, (0.64–1.34)	0.93, (0.63–1.36)
74–108	1.15, (0.80–1.66)	1.15, (0.79–1.65)	1.14, (0.79–1.65)	1.12, (0.77–1.64)
≥109	0.93, (0.64–1.36)	0.93, (0.64–1.36)	0.95, (0.65–1.40)	0.93, (0.63–1.39)
Number of pregnancies				
≤4	1, (ref)	1, (ref)	1, (ref)	1, (ref)
5	0.86, (0.59–1.27)	0.84, (0.57–1.24)	0.85, (0.58–1.26)	0.81, (0.55–1.21)
6-7	1.37, (0.98–1.90)	1.33, (0.95–1.85) ^*∗*^	1.36, (0.97–1.90)	1.36, (0.96–1.92)
≥8	0.94, (0.65–1.35)	0.92, (0.64–1.33)	0.95, (0.65–1.38)	0.95, (0.64–1.40)
Number of abortions				
0	1, (ref)	1, (ref)	1, (ref)	1, (ref)
1	1.02, (0.75–1.32)	1.01, (0.74–1.37)	1.01, (0.74–1.38)	1.08, (0.78–1.49)
≥2	0.76, (0.50–1.15)	0.76, (0.50–1.15)	0.77, (0.51–1.16)	0.79, (0.51–1.20)
Age at first living childbirth				
≤18	1, (ref)	1, (ref)	1, (ref)	1, (ref)
19-20	0.85, (0.59–1.24)	0.86, (0.59–1.24)	0.85, (0.58–1.24)	0.86, (0.58–1.26)
21–23	0.87, (0.61–1.24)	0.87, (0.61–1.24)	0.86, (0.60–1.24)	0.88, (0.60–1.28)
≥24	0.85, (0.58–1.24)	0.84, (0.57–1.23)	0.82, (0.56–1.23)	0.82, (0.55–1.22)
Age at first pregnancy				
≤17	1, (ref)	1, (ref)	1, (ref)	1, (ref)
18–20	1.00, (0.71–1.41)	1.00, (0.71–1.41)	0.97, (0.69–1.37)	1.04, (0.72–1.5)
21-22	0.88, (0.59–1.32)	0.88, (0.58–1.32)	0.87, (0.57–1.31)	0.88, (0.57–1.35)
≥23	0.90, (0.61–1.31)	0.88, (0.60–1.)	0.86, (0.58–1.26)	0.92, (0.61–1.38)
Menarche age				
≤12	1, (ref)	1, (ref)	1, (ref)	1, (ref)
13	0.99, (0.69–1.42)	0.97, (0.67–1.39)	0.99, (0.69–1.43)	1.04, (0.70–1.52)
14-15	0.99, (0.71–1.37)	0.97, (0.70–1.35)	0.99, (0.71–1.38)	1.02, (0.73–1.43)
≥16	1.35, (0.79–2.31)	1.33, (0.73–2.27)	1.31, (0.76–2.25)	1.35, (0.77–2.36)

The baseline model is stratified on the lifetime hormonal fluctuations. The adjusted model 1 is adjusted for the confounding variable age (continuous variable). The adjusted model 2 has an additional adjustment for the confounding variables related to lifestyle (cigarette and hookah smoking), history of diabetes (yes/no), history of hypertension (yes/no), BMI (continuous variable), and WSI (continuous variable). The adjusted model 3 has an additional adjustment for brushing frequency (categorical variable). ^*∗*^ Statistically significant (*p* < 0.05).

## Data Availability

The datasets used during the current study are available on the Persian Adult Cohort Study Center, Rafsanjan University of Medical Sciences, Iran. The data are not available publicly. However, upon a reasonable request, the data can be obtained from the authors.
